# The heterogeneous effects of exchange rate and stock market on CO2 emission allowance price in China: A panel quantile regression approach

**DOI:** 10.1371/journal.pone.0220808

**Published:** 2019-08-13

**Authors:** Xiaojian Su, Chao Deng

**Affiliations:** 1 School of Finance, Guangdong University of Foreign Studies, Guangzhou, P.R., China; 2 Southern China Institute of Fortune Management Research (IFMR) Guangzhou, P.R., China; Shandong University of Science and Technology, CHINA

## Abstract

This paper studies the heterogeneous effects of exchange rate and stock market on carbon emission allowance price in four emissions trading scheme pilots in China. We employ a panel quantile regression model, which can describe both individual and distributional heterogeneity. The empirical results illustrate that the effects of explanatory variables on carbon emission allowance price is heterogeneous along the whole quantiles. Specifically, exchange rate has a negative effect on carbon emission allowance price at lower quantiles, while becomes a positive effect at higher quantiles. In addition, a negative effect exists between domestic stock market and carbon emission allowance price, and the intensity decreasing along with the increase of quantile. By contrast, an increasing positive effect is discovered between European stock market and domestic carbon emission allowance prices. Finally, heterogeneous effects on carbon emission allowance price can also be proved in European Union Emission Trading Scheme (EU-ETS).

## Introduction

With the development of economies, the carbon dioxide emission has increased rapidly in decades [1], which caused many people have intensified concern about the natural environment. One hundred eighty-three countries adopted the Kyoto Protocol in 2009, designed to promote development of carbon trading markets. This agreement is designed to contribute to the improvement of such markets while helping, as a result, to protect the environment through controlling the emission of greenhouse gases. Therefore, the study on the CO_2_ emission allowance trading market has boomed over last decades. Generally speaking, its research can be divided into two categories. The first type of literature is studying price discovery, price drivers and price volatility structure of carbon emission allowances’ spot and futures markets. Previous studies that mainly used linear methods to discuss the carbon trading price have been challenged strongly by the rationality of applying nonlinear approach [2,3]. Another strand of the literature has been dedicated to exploring the drivers of CO_2_ emission allowance markets. For instance, Barros, Gilalana and De Gracia[1] explored the relationship between energy market and CO_2_ emissions, and Mansanet-Bataller and Pardo [4] demonstrated that energy price and extreme temperature are important factors in determining the price of CO_2_ emission allowance. Alberola, Chevallier and Chèze [5] indicated the effect of certain variables on carbon trading price, such as stock market index and exchange rates. Hammoudeh, Nguyen and Sousa [6] examined the influence of oil, coal, natural gas and electricity prices’ changes on CO2 emission allowance price by employing a Bayesian Structural VAR model. Chevallier [7] suggested that stock market has a negative effect on carbon emission allowance price, while Zhu, Ye, Han, Wang, He, Wei and et al. [8] found that stock index had a positive driving force to carbon price. Boutabba and Lardic [9] considered that carbon price and exchange rate are both positive and statistically significant at some periods, which suggested that exchange rate might positively relate to carbon emission allowance price.

Nowadays, China has become the biggest national carbon emitter in the world. Since 2013, Seven pilot provinces, namely, Shenzhen, Beijing, Guangdong, Shanghai, Tianjin, Hubei and Chongqing, have formally launched the carbon emissions trading schemes. As of December 31, 2016, the total trading volume of the seven pilot carbon emission trading platforms reached 86.1 million tons, and the trading volume reached 2 billion Yuan. Some work also concentrates the drivers of CO_2_ emission allowance price in China (e.g. [10–12]). However, all these studies only focus the drivers of CO_2_ emission allowance price in the single pilot province, there has been little research focus the whole of the China's carbon emissions trading market. Along those lines, this paper aims to study the effect of exchange rate and stock market on the carbon emission allowance market in four major provinces in China.

The contributions of this paper are summarized as follows. Firstly, we apply the panel quantile regression approach to analyze the heterogeneous effect of exchange rate and stock market on the carbon emission allowance market at different levels of carbon emission allowance price. Then, we find that the exchange rate has a negative effect on carbon emission allowance price except for higher quantiles, and becomes a positive effect at higher quantiles. Furthermore, stock market has a negative effect on carbon emission allowance price, and the intensity decreasing with higher quantiles. By contrast, an increasing positive effect is found between European stock market and domestic carbon emission allowance prices. Finally, we take a comparison between Chinese and European markets as a robust test. And attempts to draw more credible and widely applicable conclusions about the effect on carbon emission allowance markets.

The rest of this paper is organized as follows: The data and methodology are discussed in Section 2. The analysis of the empirical results is provided in Section 3. The paper concludes with policy recommendations in Section 4.

## Data and methodology

### Data

This paper investigates the effects of exchange rate and stock market on carbon emission allowance price, and control the energy price as control variable. We conduct an empirical analysis based on the daily time series covering panel data from four carbon emission allowance trading scheme pilots in China during the period of 2014–2019. Because Chinese carbon emission allowance trading market pilots started in 2013, and different carbon trading pilots did not have the same sample trading period over all the time, the sample period starts in 2014 after aligning four pilots trading dates. Furthermore, in order to ensure sufficient data in sample, the pilot cities without sufficient sample data were deleted, and finally Beijing, Shanghai, Hubei and Guangdong remained.

**Fig 1:**
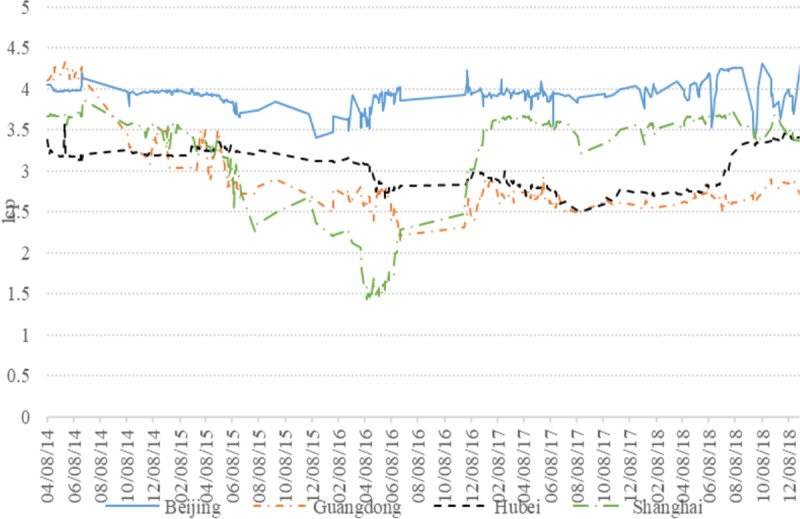
The fluctuation of carbon emission allowance prices.

Carbon emission allowance price is the dependent variable, which is denoted the closing price in the subject provinces on the China Emissions Exchange from the China Stock Market & Accounting Research Database (CSMAR). The closing prices of the European carbon trading futures market in Euros from the Investing.com database. Fig 1 shows the time series plots of the logarithmic data of carbon emission allowance prices in four provinces during 2014–2019. From Fig 1, it can observe that carbon emission allowance prices in the four provinces (including Beijing, Guangdong, Hubei and Shanghai) had a high frequency of fluctuations, while their fluctuations’ directions are similar.

From the former study, it suggests that exchange rate, stock market and energy market have relationships on carbon emission allowance price. Thus, the independent variables include exchange rate and stock market index, and the control variable is energy price. Because one of the carbon emission allowance trading markets is dominant by European, exchange rate is represented by Currency Chinese Yuan (CNY) units per EUR. The stock market index is represented by the domestic Chinese CSI 300 index [13] and, as for foreign (European) stocks, by the STOXX 600 index [14] on Investing.com. As coal is primary energy in the Chinese energy structure [15], energy price is represented by the closing price of Coking coal futures in Dalian Commodity Exchange (DJMc1), while the closing price of Brent Oil Futures (LCOJ9) is stand for foreign energy market from Investing.com [16]. Exhaustive variable descriptions are presented in Table 1.

**Table 1:** Variables’ definition.

Variable		Definition	Source
Carbon emission allowance price	lcp	The closing price of carbon emission allowance markets in China and logarithmic data	CSMAR database
	lcef	The closing price of carbon emission allowance futures market in Euros and logarithmic data	Investing.com
Exchange rate	ler	CNY units per EUR and logarithmic data	
	lier	EUR units per CNY and logarithmic data	
Stock market index	lhs300	Stock index in Shanghai and Shenzhen stock markets (CSI300) and logarithmic data	
	leu600	Stock index in European stock market (STOXX600) and logarithmic data	
Energy price	ljmqh	The closing price of Coking coal futures in Dalian Commodity Exchange (DJMc1) and logarithmic data	
	lbop	The closing price of Brent Oil Futures (LCOJ9) and logarithmic data	

### Methodology

There are few literatures that have explored the heterogeneous effect on carbon emission allowance price by panel data, so the aim of this paper is to test whether the heterogeneous effect exists across different pilots in China. From the review of previous study, using the quantile regression method is a better way to exam the heterogeneous effect [11,17,18]. Therefore, to investigate the heterogeneous effects of exchange rate and stock market on carbon emission allowance price in China, this paper employs a fixed effect panel quantile regression model. Some literatures had focus on the econometric model of applying panel quantile regression (such as [19,20]), and its form given as follows:

$$Q_{y_{it}}(\tau_{k}|\alpha_{i},x_{it})=\alpha_{i}+x_{it}^{\prime }\gamma \left(\tau_{k}\right)$$ (1)

The fixed effect panel quantile regression’s main problem is that it includes numerous fixed effects (α_i_) which caused it vulnerable to random parameters [21]. If *i*→∞, the estimator is inconsistent, while the observed measurements of each cross section are fixed. Due to the problem of the unnoticed fixed effects in quantile regression [22], Koenker [19] proposed a penalty term *λ* in the minimization to estimate numerous parameters. These parameters are estimated as follows:

$$(\hat{\gamma }\left(\tau_{k},\lambda \right),{\left\{\alpha_{i}\left(\lambda \right)\right\}}_{i=1}^{N})=\mathrm{argmin}\sum_{k=1}^{K}\sum_{t=1}^{T}\sum_{i=1}^{N}w_{k}\rho_{\tau_{k}}\left(y_{it}-\alpha_{i}-x_{it}^{\prime }\gamma \left(\tau_{k}\right)\right)+\lambda \sum_{i=1}^{N}\left|\alpha_{i}\right|$$ (2)

where *i* denotes the pilot province (*N*), *T* represents the dates of samples in every pilot province, *K* denotes the quantiles, *x* represents the matrix of independent variables and $\rho_{\tau_{k}}$ signifies the quantiles loss function. In addition, *w*_*k*_ means the *k*-th quantile’s relative weight that contributes the estimation of fixed effects at the *k*-th quantile, and this paper employs equal weighted quantile *w*_*k*_ = 1/*k* [23]. *λ* is the tuning parameter, which can decline the individual effects to zero to optimize the estimation of *β*. When *λ* = 0, the penalty term disappears and model gets the usual fixed effects estimator. The model would obtain an estimator without individual effects when *λ*→∞.

For the purpose of this paper, the Eq (2) can be modified as follows:

$$Q_{\mathrm{lcp}it}=\alpha_{i}+\xi_{t}+\gamma_{1\tau }ler_{it}+\gamma_{2\tau }lhs300_{it}+\gamma_{3\tau }leu600_{it}+\gamma_{4\tau }ljmqh_{it}+\gamma_{5\tau }lbop_{it}$$ (3)

where the pilot provinces in China are represented by *i*, and time by *t*.

## Empirical result and analysis

### Descriptive statistic

To discuss the variables in more detail, descriptive statistics are provided below in Table 2. The results show that the variables’ mean and median are close, and their skewness is close to 0 and kurtosis close to 3, which means that their distributions are near normal distribution. In Table 3, we can conclude that the absolute values of all variables’ Pearson correlation coefficients are smaller than 0.8, which has been argued in other paper, such as Mukaka [24], that when the Pearson correlation coefficient is smaller than 0.8, the two variables are not strongly correlated. Therefore, it means that these variables are not strongly correlated and the regression results are reliable.

**Table 2:** Summary statistics.

Variable	Obs	Mean	Std.Dev.	Min	Q1	Mid.	Q3	Max	Skew.	Kurto.
**lcp**	1528.00	3.27	0.58	1.44	2.79	3.27	3.74	4.34	-0.44	2.90
**lcef**	1528.00	2.02	0.44	1.47	1.67	1.91	2.07	3.21	1.08	3.09
**ler**	1528.00	2.01	0.06	1.88	1.99	2.01	2.05	2.16	0.22	3.05
**ljmqh**	1528.00	6.87	0.34	6.34	6.57	6.83	7.17	7.48	0.08	1.51
**lbop**	1528.00	4.12	0.27	3.42	3.92	4.04	4.30	4.75	0.64	3.09
**leu600**	1528.00	5.91	0.07	5.73	5.84	5.93	5.96	6.03	-0.33	1.83
**lhs300**	1528.00	8.13	0.20	7.66	8.06	8.15	8.24	8.58	-0.55	3.75

**Table 3:** Pearson correlation coefficient table.

	lcp	lcef	ler	lhs300	leu600	ljmqh	lbop
**lcp**	1.0000						
**lcef**	0.1002	1.0000					
**ler**	0.1836	0.1846	1.0000				
**lhs300**	-0.1403	0.1629	-0.7313	1.0000			
**leu600**	0.0631	0.2087	-0.4056	0.7254	1.0000		
**ljmqh**	0.0817	0.2223	0.2476	0.1147	0.2949	1.0000	
**lbop**	0.3488	0.4103	0.6231	-0.4054	-0.0071	0.1023	1.0000

### Panel quantile regression result

We proceed to the OLS panel regression and estimate results at random, fixed and pooled effects. The regression results show as Table 4.

**Table 4:** Empirical result of OLS regression.

VARIABLES	Random effect	Fixed effect	Pooled effect
**ler**	-1.494^***^	-1.494^***^	-1.494^***^
**ljmqh**	0.118^***^	0.118^***^	0.118^**^
**lbop**	0.759^***^	0.759^***^	0.759^***^
**leu600**	1.253^***^	1.253^***^	1.253^***^
**lhs300**	-0.671^***^	-0.671^***^	-0.671^***^
**Constant**	0.393	0.393	0.393
**Observations**	1,528.000	1,528.000	1,528.000
**Hausman Test:** H_0_: difference in coefficients not systematic			

Note

*** and ** denote statistical significance at the 1% and 5% levels respectively^.^; the dependent variable is denoted by lcp.

Because the Hausman test’s chi2 < 0, we choose the random effect results to interpret the relationship between variables. As the empirical results show, this paper concludes that all independent variables are related significantly to the dependent variable. The results demonstrate that the exchange rate as defined has a negative relationship with carbon emission allowance price. Domestic stock indices by contrast have an inverse relationship to carbon emission allowance prices, while the foreign stock market index had a direct, positive relationship with such prices.

From Table 5, we can conclude that carbon emission allowance price is negatively affected by exchange rate at middle or lower quantiles. It is significant in the opposite direction at the 95^th^ percentile and higher quantiles, which finding shows the same association between exchange rate and carbon emission allowance price [9]. As to stock markets, domestic markets are significantly negatively associated with carbon emission allowance prices for most of the quantiles which is consistent with the findings of Chevallier [7], but the foreign stock market has a significantly positive effect on carbon trading prices at 80^th^ and lower quantiles. Fluctuations in energy market also have complex and varied effects on carbon emission allowance market. For the domestic market, there is a significant positive relationship between the two at the 60^th^ and lower quantiles, which changes to a significant negative relationship at the 90^th^ and higher quantiles. But the foreign energy market has a significant positive effect on Chinese carbon emission allowance prices across all quantiles.

**Table 5:** Empirical results of panel quantile regression.

variables	Quantiles levels										
	Q_0.05_	Q_0.1_	Q_0.2_	Q_0.3_	Q_0.4_	Q_0.5_	Q_0.6_	Q_0.7_	Q_0.8_	Q_0.9_	Q_0.95_
**ler**	-5.000^***^	-4.021^***^	-3.225^***^	-2.517^***^	-1.942^***^	-1.435^***^	-0.961^***^	-0.373	0.031	0.619	1.198^**^
**lhs300**	-1.079^***^	-0.965^***^	-0.872^***^	-0.790^***^	-0.723^***^	-0.664^***^	-0.609^***^	-0.540^***^	-0.494^***^	-0.425^***^	-0.358^*^
**leu600**	2.557^***^	2.193^***^	1.897^***^	1.634^***^	1.420^***^	1.231^***^	1.055^***^	0.837^***^	0.686^**^	0.468	0.253
**ljmqh**	0.500^***^	0.393^***^	0.307^***^	0.229^***^	0.167^***^	0.111^***^	0.060^*^	-0.005	-0.049	-0.113^**^	-0.176^***^
**lbop**	1.097^***^	1.002^***^	0.926^***^	0.858^***^	0.802^***^	0.753^***^	0.708^***^	0.651^***^	0.612^***^	0.556^***^	0.500^***^

Note

***, **, and * denote statistical significance at the 1%, 5% and 10% levels respectively^.^; the dependent variable is denoted by lcp.

Because of the logarithmic data, all the empirical results are estimating elasticities of dependent and independent variables, which can be interpreted as: A unit of the dependent variable will change by an equivalent percentage when a unit of the independent variable changes by 1%, with other conditions unchanged.

**Fig 2:**
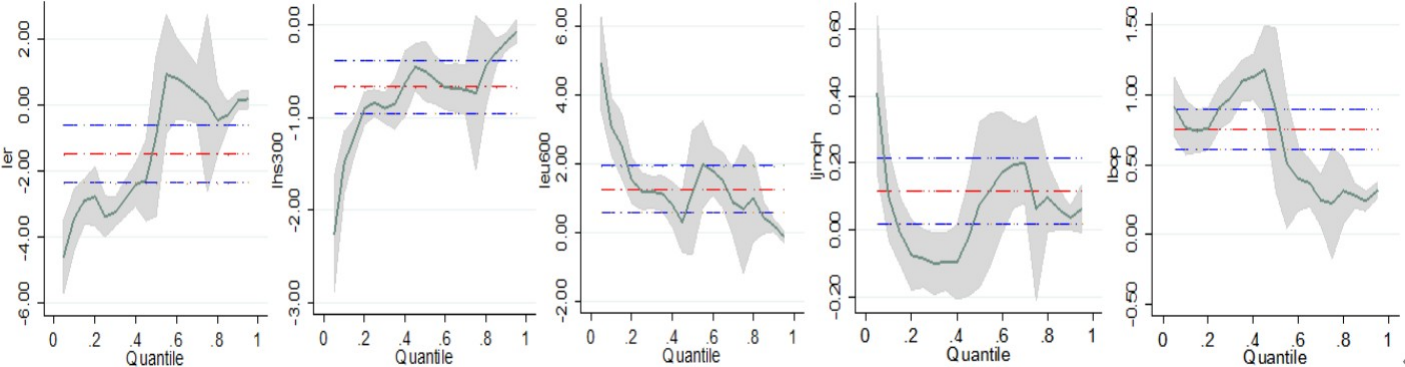
Empirical result of multiple variables on panel quantile regression. Note: Quantile regression estimates with 95% confidence intervals for the effects of influential factors on carbon emission allowance prices. The vertical axes show the coefficient estimates of the variables over the carbon emission allowance prices' distribution. The horizontal axes depict the quantile levels. The red horizontal dash doc lines represent the OLS estimations with their 95% confidence interval (blue dashed doc line).

From Fig 2, we can see the trend of coefficient changes by quantile. Unlike the OLS estimator, the results of panel quantile regression can estimate the heterogeneous effects of exchange rates and stock market on carbon emission allowance prices. These results could also provide more details about the carbon emission allowance market and suggest possible modifications of policy in operating this market. The trend of coefficient variables is fluctuating round the OLS estimator. We can claim that exchange rate has a positive effect on carbon prices at high quantiles, instead of a negative one as in the OLS estimates.

### Comparison with European market

From the previous empirical results, our research concludes that exchange rates and stock index have a significant nonlinear relationship with carbon emission allowance price on Chinese carbon emission allowance trading market. To test the validity of this conclusion, we change the dependent variable, using the European carbon emission allowance futures price. Because we only have time-series data without cross-section data for European trading futures prices, we cannot use panel quantile regression. For convenience, this paper employs the quantile regression (e.g. [25]) to analyze the impact of exchange rate and stock market on European carbon emission allowance market and. Also, the study changes the exchange rate to CNY/EUR and logarithmic data.

**Fig 3:**
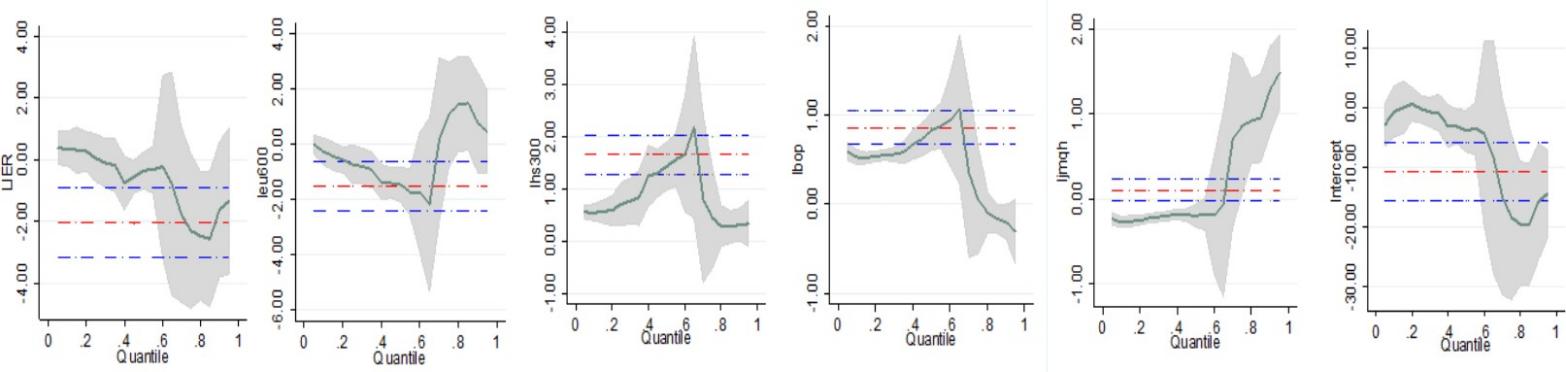
Empirical result of European market. Note: Quantile regression estimates with 95% confidence intervals for the effects of influential factors on carbon emission allowance prices. The vertical axes show the coefficient estimates of the variables over the carbon emission allowance prices' distribution. The horizontal axes depict the quantile levels. The red horizontal dash doc lines represent the corresponding OLS estimations with their 95% confidence interval (blue dashed doc line).

From the comparison with heterogeneous results in China’s carbon emission allowance price, Table 6 and Fig 3 show almost the same findings in European carbon emission allowance market prices, which would support the conclusion in the previous results. For example, when the exchange rate CNY appreciates which means the exchange rate EUR depreciates, the exchange rate CNY has a negative relationship with Chinese carbon emission allowance market, while exchange rate EUR also admits negative impact on European carbon emission allowance price. Furthermore, European stock market has the same effect as in the previous case. That is, domestic carbon emission allowance price is significantly negatively influenced by domestic stock market, and a positive effect for foreign stock market.

**Table 6:** Empirical result of European market.

variables	Quantiles levels										
	Q_0.05_	Q_0.1_	Q_0.2_	Q_0.3_	Q_0.4_	Q_0.5_	Q_0.6_	Q_0.7_	Q_0.8_	Q_0.9_	Q_0.95_
**ler**	0.386	0.364	0.273	-0.101	-0.763	-0.349	-0.229	-1.773	-2.469^**^	-1.620	-1.341
**lhs300**	0.553^***^	0.547^***^	0.583^***^	0.764^***^	1.244^***^	1.433^***^	1.669^**^	0.805	0.287	0.314	0.335
**leu600**	0.005	-0.272	-0.575^*^	-0.795^**^	-1.382^***^	-1.453^***^	-1.736^*^	0.170	1.474	0.762	0.457
**ljmqh**	-0.232^***^	-0.272^***^	-0.252^***^	-0.212^***^	-0.179^***^	-0.195^***^	-0.184	0.698	0.920^***^	1.285^***^	1.489^***^
**lbop**	0.592^***^	0.529^***^	0.540^***^	0.562^***^	0.675^***^	0.826^***^	0.945^***^	0.350	-0.094	-0.198	-0.308
**_cons**	-2.902	-0.705	0.471	-0.788	-3.207	-3.962^*^	-4.467	-15.060	-19.560^***^	-15.850^**^	-14.530^***^

Note

***, **, and * denote statistical significance at the 1%, 5% and 10% levels respectively^.^; the dependent variable is denoted by lcef.

Summarizing the comparison between Chinese and European carbon emission allowance trading market, we can conclude that the heterogeneous effect of exchange rate and stock are similar to the carbon emission allowance price, such as exchange rate has negative effect on domestic carbon trading market when the currency value is higher, positively when the currency value is lower. Besides, the domestic stock market is negatively affecting the price of domestic carbon emission allowance, while effect of the foreign stock market is positive.

### Empirical result analysis

**Fig 4:**
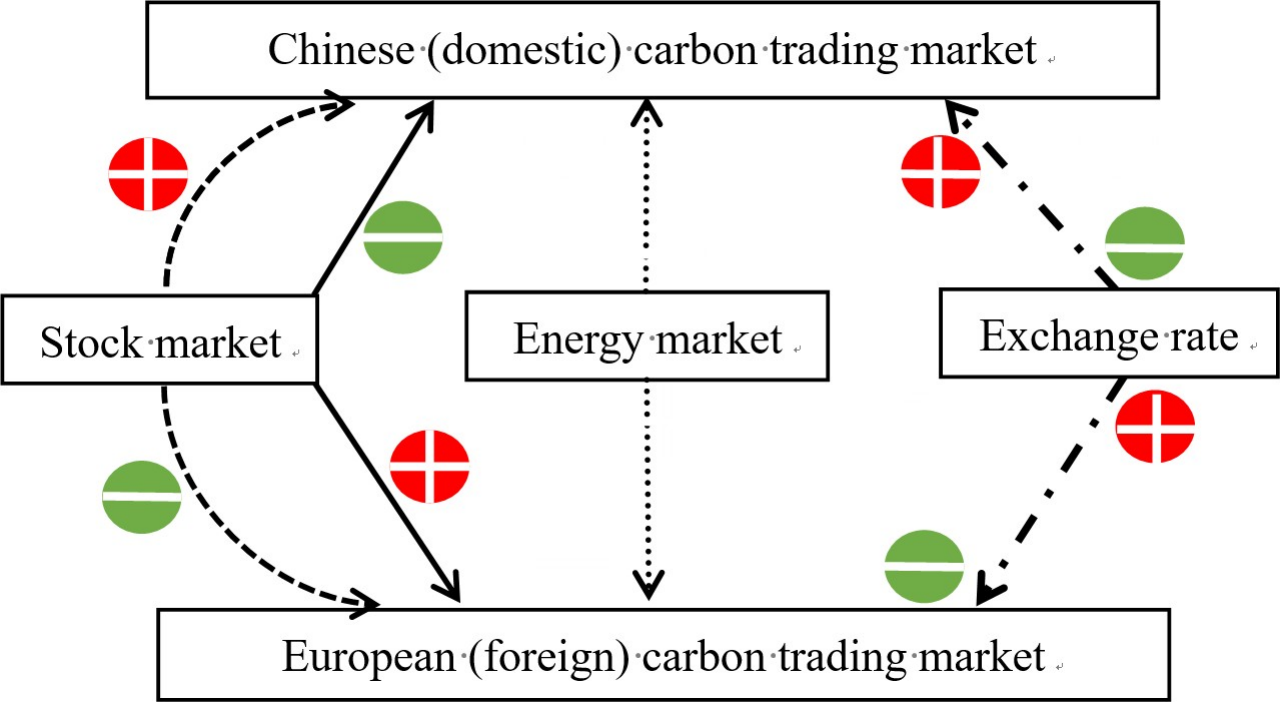
The effects of independent variables on dependent variables. Note: solid lines and dash lines represent Chinese markets (stock market and energy market) and European markets (stock market and energy market), respectively. Dashed doc line represents exchange rate. The doc line represents the control variable—energy market. Green circle and red circle mean negative and positive relationships, respectively, with carbon emission allowance market price. Half-line refers to variable relationships changing from low to high quantile.

Fig 4 describes the heterogeneous effects of the exchange rate and stock market on Carbon trading price. From Fig 4, we can conclude that the exchange rate of a country exhibits a negative effect on that country’s domestic carbon emission allowance market price. For the case of lower carbon emission allowance price, following the law of supply and demand, which suggests that the supply in the domestic market is fulfilled and need to broaden the foreign carbon emission trading market. At lower exchange rate means the higher CNY currency value that reduces the demand of carbon emission allowance in foreign market, so the carbon emission allowance price is lower. In other words, exchange rate shows a negative impact on lower carbon emission allowance price. At higher carbon emission allowance price, the finding is that the exchange rate becomes a positive driving force for carbon emission allowance trading market, which is similar with the result of Boutabba and Lardic [9]. The reason may be the demand of carbon emission trading in foreign market raises when the CNY currency value depreciates.

The domestic stock market has a significantly negative effect on domestic carbon emission allowance trading market which finding is like the discovery of Chevallier [7], but differently, with the increase of carbon emission allowance price, its intensity is gradually decreasing. The reason caused negative effect might be that the financing function of Chinese carbon emission allowance trading market has increased in recent years, while there is a capital competitive relationship with stock market. In other words, when carbon emission allowance price at lower quantiles, it might suggest that the pricing mechanism of carbon emission allowance trading market was not perfect. As a result, it is easily influenced by the stock market. Besides, as a new financing instrument of capital market in China, the financing capacity of carbon emission allowance trading market is squeezed by stock market which is a more perfect capital market in China. Consequently, carbon emission allowance market is strongly negatively impacted by the domestic stock market at lower quantiles. When turns to higher quantiles, the pricing mechanism is mature and the negative effect of stock market is weaker.

As for foreign stock, it has significantly positive impact on carbon emission allowance trading market while the impact is getting weaker and weaker across quantiles. Because of the imperfect pricing mechanism of carbon emission allowance market, it is an easy arbitrage at lower quantiles for foreign capital market, such as stock market. Therefore, it has a positive influence on the carbon emission allowance price at lower quantiles. The arbitrage opportunities decrease when carbon emission allowance price is at higher quantiles. Thus, the effect of foreign stock market on carbon emission allowance price becomes weaker at higher quantiles.

## Conclusions

The objective of this research is to study the heterogeneous effects of exchange rate and stock market on carbon emission allowance trading market from the perspective of panel quantile regression. This approach characterizes the heterogeneous effects of the changes in exchange rate and stock market on both normal and extreme distributions of carbon emission allowance price. From the empirical evidences, it demonstrates that exchange rate has a heterogeneous impact on carbon emission allowance price. For the currency exchange rate (CNY), involving a negative impact into a positive impact with higher quantiles in the Chinese carbon market. For the stock market, we can find that the domestic stock market has a negative effect with carbon emission allowance trading market, and the degree of the effect weakening with the increasing of the stock market index. But European stock market has positive effect on Chinese carbon emission allowance trading market, while negatively affect European carbon emission allowance market.

## Supporting information

S1 File Raw data of 4 pilots of Chinese carbon emission allowance trading market and European carbon emission allowance trading market for 2014–2019. In additional, the raw data of exchange rate, stock market and energy market are showing in the file.
(XLSX)

S2 File Accessible channels of all original data in the paper are showing in this file. (DOCX)
