# Supplementary material for: The heterogeneous effects of exchange rate and stock market on CO2 emission allowance price in China: A panel quantile regression approach
Source: PLoS One. 2019 Aug 13;14(8):e0220808. doi: 10.1371/journal.pone.0220808 (PMC6692012; doi:10.1371/journal.pone.0220808)
Supplement: S2 File — (DOCX) [file pone.0220808.s002.docx]

**Data Availability**

cp, please visit: (CSMAR database)

<http://www.gtarsc.com/SingleTable/DataBaseInfo?nodeid=34763>

（Detail way to get the specific data from the general CSMAR website:

First choose the “Data Center”, then choose the “Green Economy Series”, next choose the “Global Warming” index, then choose the “Carbon Emissions Trading” index and turn to “China Carbon Emissions Trading Information Sheet (Daily)”, finally set the fields, download the data. Data underlying the study are third-party data as described in the Supporting Information file S2 File. The authors confirm they did not have any special access to this data which other researchers would not have.）

lcp=ln(cp);

cef, please visit: (Investing.com)

<https://cn.investing.com/commodities/carbon-emissions-historical-data>

lcef=ln(cef);

er, please visit: (Investing.com)

<https://cn.investing.com/currencies/eur-cny-historical-data>

ler=ln(er);

ier, please visit: (Investing.com)

<https://cn.investing.com/currencies/cny-eur-historical-data>

lier=ln(ier);

hs300, please visit: (Investing.com)

<https://cn.investing.com/indices/csi300-historical-data>

lhs300=ln(hs300);

eu600, please visit: (Investing.com)

<https://cn.investing.com/indices/stoxx-600-historical-data>

leu600=ln(eu600);

jmqh, please visit: (Investing.com)

<https://cn.investing.com/commodities/coking-coal-futures-historical-data>

ljmqh=ln(jmqh);

bop, please visit: (Investing.com)

<https://cn.investing.com/commodities/brent-oil-historical-data>

lbop=ln(bop).
